# Immunomodulatory Action of Substituted 1,3,4-Thiadiazines on the Course of Myocardial Infarction

**DOI:** 10.3390/molecules23071611

**Published:** 2018-07-02

**Authors:** Alexey P. Sarapultsev, Pavel M. Vassiliev, Petr A. Sarapultsev, Oleg N. Chupakhin, Laura R. Ianalieva, Larisa P. Sidorova

**Affiliations:** 1 Institute of Immunology and Physiology of the Ural Branch of RAS, Pervomayskaya 106, Ekaterinburg 620049, Russia; p.sarapultsev@gmail.com; 2 Department of Pharmacology and Bioinformatics, Volgograd State Medical University, Pavshikh Bortsov Square 1, Volgograd 400131, Russia; Pvassiliev@mail.ru (P.M.V.); yanalieva.laura@yandex.ru (L.R.I.); 3 The IJ Postovsky Institute of Organic Synthesis of the Ural Branch of RAS, Akademicheskaya/S. Kovalevskoi, 22/20, Ekaterinburg 620990, Russia; chupakhin@ios.uran.ru; 4 Ural Federal University named after the First President of Russia B. N. Yeltsin, 19 Mira Street, Ekaterinburg 620002, Russia; vlapp@isnet.ru

**Keywords:** 1,3,4-thiadiazines, immunomodulators, myocardial infarction, PI3K-AKT signaling pathway, stress response

## Abstract

This review focuses on the biological action of the compounds from the group of substituted 1,3,4-thiadiazines on stress response and myocardial infarction. The aim of this review is to propose the possible mechanisms of action of 1,3,4-thiadiazines and offer prospectives in the development of new derivatives as therapeutic agents. It is known, that compounds that have biological effects similar to those used as antidepressants can down-regulate the secretion of proinflammatory cytokines, up-regulate the release of anti-inflammatory ones and affect cell recruitment, which allows them to be considered immunomodulators as well. The results of pharmacological evaluation, in silico studies, and in vivo experiments of several compounds from the group of substituted 1,3,4-thiadiazines with antidepressant properties are presented. It is proposed that the cardioprotective effects of substituted 1,3,4-thiadiazines might be explained by the peculiarities of their multi-target action: the ability of the compounds to interact with various types of receptors and transporters of dopaminergic, serotonergic and acetylcholinergic systems and to block the kinase signal pathway PI3K-AKT. The described effects of substituted 1,3,4-thiadiazines suggest that it is necessary to search for a new agents for limiting the peripheral inflammatory/ischemic damage through the entral mechanisms of stress reaction and modifying pro-inflammatory cytokine signaling pathways in the brain.

## 1. Introduction

It has been well recognized that the course of many diseases, the pathogenesis of which involves changes in the immune system, can be modified by the administration of biologicals or chemicals that activate or suppress key pathways in the immune system. However, in the medical literature, one finds multiple and conflicting definitions for the terms immunosuppression and immunomodulation depending on the author, publication, or agency defining these terms [1]. According to Lebish (1987), immunomodulators are “those intrinsic or extrinsic substances which regulate or alter the scope, type, duration or competency of the immune response” [2]. The term “regulation”, in this or similar definitions, always includes the ability of substances to upregulate or downregulate specific aspects of the host response [3]. The properties of immunomodulators were limited only to the restoration of immune system functions in some later definitions. According to this point of view, immunomodulators are medicines that restore the functions of the immune system in therapeutic doses (effective immune defense), by lowering the increased and increasing the lowered immunity [4]. Moreover, some researchers even categorize agents based on the most clinically relevant data, which are the documented occurrences of immune-mediated, clinically relevant complications that are compiled from the actual human data [1,2,3,4,5]. According to them, immunomodulators refer to those agents that modulate the immune response to control inflammatory disease activity without being associated with a statistically significant increased risk of clinically manifested immune-mediated complications [1]. The direction of an immunomodulator’s action (and the previous state of the immune system), with that definition, is not mentioned at all. Finally, according to the U.S. Food and Drug Administration (FDA) guidelines, immunomodulators are the non-vaccine and non-allergenic products intended to treat disease by inhibiting or down-regulating a pre-existing, pathological immune response [5]. Thus, the later definition postulated not only the direction of action (inhibiting or down-regulating) and the state of immune response (the exaggerated), but also the chemical nature of compounds, separating them from vaccines, antibodies, enzymes and cytokines. The FDA’s definition is mostly clear, although it is not without shortcomings, as it excludes some possible mechanisms of action by these agents (described by Lebish, 1987), such as increasing the ability of the host to tolerate damage by cytotoxic modality, augmenting and/or restoring effector mechanisms or mediators of host defense, or replacing the damaged effector or mediator mechanisms [2]. Moreover, the possible links between the immune response and stress reaction, which often accompany the disturbances of the former, also slip out of sight.

Selye, in 1936, defined stress physiologically as the state in which the sympathoadrenomedullary system and the limbic-hypothalamic-pituitary-adrenal axis (HPA) are coactivated [6]. Recently, the understanding of the interactions between the HPA axis and inflammatory reactions mediated by the immune system has expanded greatly, with many studies showing that stress can affect various parts of the cellular immune response [7]. Experimental and clinical studies have shown that both artificial and natural stressors alter the activity of cellular affecters of the immune system (lymphocytes and macrophages), and these changes depend on the type of immune response, the physical and psychological characteristics of the stressor, the duration of its action, and also on the initial state of the immune system [8]. With that, according to the traditional interpretation of stress-immune interactions, the immunosuppressive nature of the stress response is an example of the harmful, pathological effects of stress on the body [9].

However, more recently this interpretation has changed, and some stress hormone effects are now interpreted as an adaptive redistribution of immune resources [10,11,12]. According to Adamo, (2017) the stress response and the immune system should be viewed not as independent entities, but as different parts of a larger defensive system [10]. This defensive system is itself embedded within the web of the animal’s total physiological network. Some network pathways are shared between the stress and immune responses [10]. This sharing can lead to both conflict and cross-tolerance. Under some conditions, network pathways are also borrowed between the two responses, and some pathways become reconfigured [10]. Stress hormones, for example, help reconfigure immune resources to enhance protection against wound infections during fight-or-flight behavior [11]. Even the chronic effects of stress hormones are context-dependent; immunosuppression is not necessarily the outcome [13]: many animals have evolved under a chronic threat of predation, which led to the increased stress levels, but those elevated levels do not appear to reduce the disease resistance [10].

That is why the neuroendocrine and immune systems have common signal mediators and receptors, while the brain has an immunoregulatory role, and the immune system has a sensory function [14]. This finds confirmation in the results of in vivo and in vitro experiments, where the neurotransmitters such as norepinephrine, serotonin, dopamine, and acetylcholine; neuropeptides such as enkephalins, substance P, vasoactive intestinal peptide, corticotrophin-releasing factor, and neuropeptide Y; neurohormones such as growth hormone, adrenocorticotropin hormone, and prolactin; and adrenal hormones such as corticosteroids and epinephrine, affect the immune functions, while the receptors for these molecules are present on surface of immune cells [7]. Among the neurotransmitters mentioned, the three cytokines—tumor necrosis factor, interleukin-1, and interleukin-6 secreted from activated immune cells are the primary agents, which can in turn change the function of the hypothalamic–pituitary–adrenal (HPA) axis. These three proinflammatory cytokines activate the HPA axis independently as well as in combination. Moreover, inflammation might also activate the HPA axis indirectly. This could occur through the stimulation of the central noradrenergic stress system by cytokines and other mediators that act first on stress-system neurons outside the blood–brain barrier (the area postrema) or on neurons inside the barrier, through the endothelial–glial–neuronal cascade mentioned above [10]. Additionally, inflammatory sites contain nociceptive, visceral, and somatosensory afferent neurons, which stimulate the noradrenergic and corticotropin-releasing hormone stress systems through an ascending spinal or cerebral nerve route [10,15]. All of these observations suggest that, since any immunomodulatory agent will affect the stress response, the substances that affect the latter might, in turn, have an immunomodulatory effect.

## 2. Materials and Methods

### 2.1. The Possibility of Influencing the Functions of the Immune System via the Central Mechanisms of the Stress Response

This view finds confirmation. According to the literature, antidepressant drugs, which affect depression and stress, were frequently shown to impact the dysregulated immune response in depressed patients [16], mostly by improving the serum cytokine profile during therapy [17,18,19], which might result from their influence on cytokine release [19,20,21,22,23] or through their action on lymphocytes and macrophages [24,25]. Moreover, due to the impact of antidepressants on serotonin turnover, it can be speculated that antidepressants might alter the immune response at the early stage of cell recruitment to the site of inflammation, which, among others, depends on platelet-derived serotonin [26]. Among the recent studies, the work of K. Nazimek et al. (2016), revealed the direct impact of various antidepressant drugs on macrophages and have shown that administration of some of them (fluoxetine, venlafaxine, and moclobemide) resulted in suppression of humoral and cell-mediated immunity with a reduction of the release of macrophage proinflammatory mediators and the expression of antigen-presentation markers [27]. Thus, compounds that have biological effects similar to those used as antidepressants can down-regulate the secretion of proinflammatory cytokines, up-regulate the release of anti-inflammatory ones [26] and affect cell recruitment, which allows them to be considered immunomodulators as well.

Many experimental and clinical studies were conducted in accordance with the evidence that stress affects the immune system and even delays wound healing [28]. Malinin et al. (2004), having summarized the available data on the history of serotonin metabolism and mechanism of action of ketanserin, suggested that the topical use of selective serotonin reuptake inhibitors (SSRIs) might represent a promising avenue for future strategies affecting wound repair in high-risk patients, especially those with diabetes mellitus, venous insufficiency, obesity, and other vascular disorders [29]. Subsequently, it was shown that fluoxetine significantly improved healing of cutaneous wounds in stressed and, to a lesser extent, in non-stressed animals and paroxetine administration enhanced wound healing by increasing the number of fibroblasts and causing better epithelialization over time in healthy but not in diabetic rats [30]. However, the negative impact of SSRIs is also described in the literature, as fluoxetine was shown to exert a direct, inhibitory effect on osteoblast differentiation and mineralization in disparate models of bone repair [31]. Thus, the clinical implications of these findings need further investigation.

Some studies also examined the effects of midazolam, a commonly used benzodiazepine, upon burn wound healing. Babcock et al. (2012) treated mice with midazolam (1 mg/kg) daily after a burn injury and observed a decrease in IL-1β, TNF-α, IL-6, IL-10, and TGF-β levels compared to saline-treated mice [32]. Another study by this group, using the same treatment with midazolam, investigated whether psychological stress (such as predator exposure) could alter survival following *Pseudomonas aeruginosa* infection [33]. The results showed that midazolam had a protective effect in mice. Together, these studies suggest that benzodiazepines can modulate the immune system and a host of inflammatory mediators, leading to a detrimental effect on healing and tissue regeneration.

The beneficial effects from pre-surgical interventions, which lowered the intensity of stress in some studies were also revealed. These beneficial effects included decreased anxiety and stress reductions when hospitalized, fewer post-operative complications, better treatment compliance, less pain and reduced use of analgesics, and alterations in various physiological indices [28,34]. Given the substantial consequences of stress, even small reductions in anxiety could have substantial clinical consequences, both directly and indirectly [28]. More broadly, researchers have used several diverse strategies to modulate immune function via influence on stress-reaction, including relaxation, hypnosis, exercise, classical conditioning, self-disclosure and cognitive behavioral interventions [28]. These interventions generally have produced positive endocrine and immune changes [35,36,37]. Although it is not yet clear to what extent these positive immunological changes translate into any concrete improvements in relevant aspects of health, such as alterations in the incidence, severity or duration of infectious and/or malignant disease, the preliminary evidence seems to be promising [28].

### 2.2. The Compounds from the Group of Substituted 1,3,4-Thiadiazines

Among the promising compounds that act like immunomodulators are the substituted 1,3,4-thiadiazines (Table 1). A variety of biological activities has been reported for many thiadiazine derivatives, such as antimicrobial [38,39,40,41], antihelmintic [42], anti-inflammatory [43,44], analgesic [45], anticancer [45,46], tuberculostatic [47] and anti-aggregant [48,49,50] activities (Table 1).

**Figure 1:**
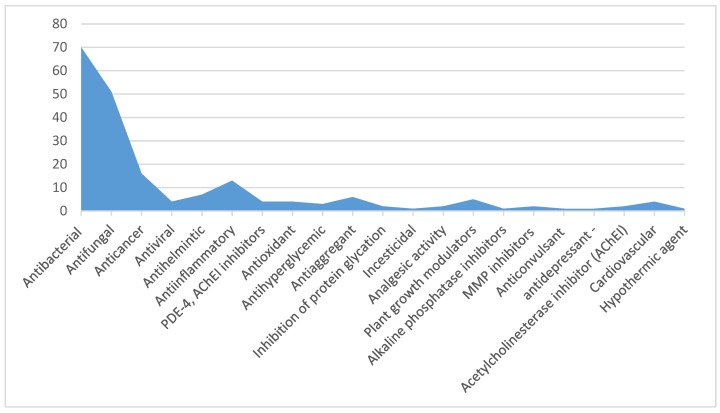
Estimated types of activity of 1,3,4-thiadiazines. Legend: the graph presents data on the types of biological activity described in the literature for 1,3,4-thiadiazines. The data may be incomplete, since they were obtained on the basis of a search query from December 2016 (1,3,4-thiadiazines AND [1,3,4]thiadiazines) in Scopus/Web of Science/Pubmed. 272 papers were found on these compounds, of which the presence/absence of biological effects was described in 132. The patents were not included in the search.

Some of the compounds are able to inhibit the non-enzymatic glycosylation of proteins in experiments in vitro [51] and to cause hypothermia in experiments in vivo [52]. Several substituted 1,3,4-thiadiazines were provided as anaesthetic, cardiovascular and hypometabolic agents [53]. With that, even the superficial analysis of published works indexed in databases Scopus, WoS and Pubmed shows that most researchers focused on the antibacterial/antifungal properties of this class of compounds (Figure 1).

### 2.3. The Action of the Compounds from the Group of Substituted 1,3,4-Thiadiazines on Myocardial Ischemia and Myocardial Infarction

In the CheMBL 24.1 database 3768 records of the compounds, the structure of which includes the 1.3.4-thiadiazine cycle, were found. However, there were no substances among them that have been investigated for the presence of any cardiotropic activity (including the myocardial ischemia and myocardial infarction). In the same database (CheMBL 24.1), 366 records, describing the experimental testing of the new substances on the model of myocardial infarction were found. Among them, only two compounds contained the substructure fragment with sulfur and nitrogen in the cycle. The chemical structures and the properties of those compounds are presented in Table 2 and Table 3.

In the PubMed and ScienceDirect search engines, no publications were published on the activity of 1,3,4-thiadiazines in myocardial infarction, other than those referenced in this review [48,49,50,51,52,53,54] (search query “thiadiazine myocardial infarction”).

Thus, the described latitude of biological activity spectrums of substituted thiadiazines, as well as the previous experimental data [53,54], serves as the basis for conducting the series of studies of the compound’s immunomodulatory properties in silico and in vivo. The aim of in silico studies is to outline the spectrum of biological activity of the compounds and to identify the potential mechanisms of action.

## 3. Experiment

### 3.1. Pharmacological Evaluation

Based on the previous data, the pharmacological evaluation in vivo and in vitro for anti-psychotic/antidepressant activity of the series of substituted 1,3,4-thiadiazines was conducted. According to the conducted pharmacological study in vivo and in vitro, the leading compound L-17 (2-morpholino-5-phenyl-6*H*-1,3,4-thiadiazine, hydrobromide), possessed the combination of adrenergic properties, as well as certain characteristics of choline and serotonin blockers and acted like the atypical antipsychotic agent Eglonyl (sulpiride) [54].

### 3.2. In Silico Studies

In silico determination of the most promising targeted activities for the leader compound (2-morpholino-5-phenyl-6*H*-1,3,4-thiadiazine hydrobromide) and the prediction of the most promising target activity spectrum for compound L-17, was performed in several stages: first, via computer program BBB Predictor 0.90 [55], the potential blood–brain barrier (BBB) penetration was evaluated followed by the use of two prediction algorithms (AdaBust, SVM) and four methods for descriptor representation of the chemical structures (MACCS, OpenbabelFP2, Molprint 2D, PubChem); the 8 pairs of scoring functions were calculated: BBB+, the ability of the compound to accumulate in the CNS, BBB−, and the alternative score. The result was a 10% truncated mean BBB +/BBB− ratio, which was 1.78 for the L-17 compound. The obtained value reflected the high ability of the L-17 compound to penetrate the BBB and, consequently, to exhibit the psychotropic properties.

Second, via the program PASS 10.4 Professional Extended [56], the prediction for the presence/absence of 480 systemic types of pharmacological activity was performed. Promising activities were those for which the calculated probability of having activity Pa was ≥0.1 and the likelihood ratio between Pa/Pi was ≥1.0, where Pi was the calculated probability of lack of activity. Taking into account the high ability of compound L-17 to penetrate the BBB and the results of its pharmacological evaluation [52], the following activities were identified for further targeted analysis: general anesthetic (Pa = 0.413, Pa/Pi = 304.33), anesthetic (Pa = 0.464, Pa/Pi = 35.69), cognition disorders treatment (Pa = 0.489, Pa/Pi = 27.17), anti-inflammatory (Pa = 0.625, Pa/Pi = 23.15), phobic disorders treatment (Pa = 0.703, Pa/Pi = 9.37), psychotropic (Pa = 0.258, P /Pi = 1.74), immunostimulant (Pa = 0.191, Pa/Pi = 1.39), antinociceptive (Pa = 0.271, Pa/Pi = 1.32), cardiovascular analeptic (Pa = 0.170, Pa/Pi = 1.21), antidepressant (Pa = 0.162, Pa/Pi = 1.14).

The prediction of the presence/absence of the linked target activities was performed during the third stage of the in silico study, for the aforementioned target systemic types of pharmacological activity, using the PharmaExpert 10.1 program [57]. According to joint predictive evaluations in PASS, PharmaExpert and experimental data [52], the most likely targeted activities corresponding to the systemic pharmacological effects of compound L-17 were found: 5-hydroxytryptamine 3 receptor antagonist (Pa = 0.139, Pa/Pi = 1.17), 5-hydroxytryptamine release inhibitor (Pa = 0.225, Pa/Pi = 1.01), acetylcholine agonist (Pa = 0.154, Pa/Pi = 7.00), benzodiazepine agonist (Pa = 0.141, Pa/Pi = 5.64), benzodiazepine omega receptor agonist (Pa = 0.385, Pa/Pi = 2.89), calpain inhibitor (Pa = 0.375, Pa/Pi = 19.74), cholinergic (Pa = 0.153, Pa/Pi = 3.26), complement factor D inhibitor (Pa = 0.430, Pa/Pi = 4.06), GABA B receptor agonist (Pa = 0.163, Pa/Pi = 1.46), GABA receptor agonist (Pa = 0.257, Pa/Pi = 6.27), glutamate release inhibitor (Pa = 0.113, Pa/Pi = 1.53), Janus tyrosine kinase inhibitor (Pa = 0.193, Pa/Pi = 6.43), Neurotransmitter uptake inhibitor (Pa = 0.470, Pa/Pi = 6.35). A separate analysis in the PASS program revealed that the L-17 compound practically did not act on the glutamatergic system (for glutamate, AMPA, NMDA and kainate receptors).

While the L-17 compound manifests itself as an atypical mild antipsychotic, antidepressant and adrenoblocker, with serotonergic, cholinergic (blocking), adrenergic (blockade of α1-adrenergic receptors), dopaminergic, GABA-ergic, and glutamatergic actions [54], for the subsequent analysis of its multitarget mechanism of action the following 15 target proteins were chosen. The list of proteins included the serotonin receptor type 3A (5-HT3A), the serotonin transporter (SERT), the muscarinic cholinergic receptor type 1 (CHRM1), the dopamine receptor type 1 (DRD1), the dopamine receptor type 2 (DRD2), the dopamine transporter (DAT), the α1-adrenoreceptor (ADRA1A), the noradrenaline transporter (NET), the α1-subunit of GABA-A receptor (GABRA1), the β2-subunit of GABA-A receptor (GABRB2), the γ2-subunit of GABA-A receptor (GABRG2),the GABA transporter of type 1 (GAT1), Janus tyrosine kinase type 3 (JAK3), the calpain 1 (CAPN1), and the complementary factor D (adipsin) CFD.

Furthermore, for a comparative evaluation of the L-17 compound’s affinity to the selected biotargets, the docking of the compound to the specific binding sites of these proteins was performed. Experimental X-ray 3D models were obtained from Protein Data Bank in Europe [58] for 5 targets and for each target protein the longest model, with the maximum resolution including an inhibitor, was chosen among all available models to allow the unambiguous determination of the binding site’s position. The models with PDB codes 5I6X [59], 5CXV [60], 3LXL [61], 1ZCM [62], 1DIC [63] for SERT, CHRM1, JAK3, CAPN1, CFD, respectively, were found. Information on the binding site for adipsin CFD was obtained from [64]. The experimental 3-D models were not available in the Protein Data Bank in Europe for 10 targets, and therefore a search for the best theoretical 3-D models from the Database of Comparative Protein Structure Models [65] was conducted. Among the available models, the longest, with the highest statistical significance, were selected, while the data on binding sites was found in available literature. The models with the following Internal ID ModBase were found (references are given to works with data about binding sites): e056050ca26ea7251f01816dc9bccde8 [66]; 28b6261001683ffd97d78ffc6fd4448a [67]; 091bc097c64a5a1726ec1a99b9228fc4 [68]; 664e40366933e7b160379446201fbf4e [69]; 0be8c685ba7d1551a3f2c7252e9c72c7 [70]; b99b37c984483657d38cd8fe9c006809 [71]; b3709eea5375f1242ba57ac4b578cded, 8e2807a55eac507927d600810aaf5386, b5b3593bf4fc198659b14bdfb9ee4ba4 [72]; 6ef1240e3cc222adef0ad22bb83881ae [73]; for 5-HT3A, DRD1, DRD2, DAT, ADRA1A, NET, GABRA1, GABRB2, GABRG2, GAT1, respectively.

The 3-D model of the L-17 compound was constructed using the molecular mechanics methods in the MarvinSketch program 15.6.15 [74], followed by optimization with the semi-empirical quantum chemical method PM7 in the MOPAC2016 program [75]. The docking was performed using AutoDockVina 1.1.2 [76], five times per target, including calculating the minimum binding energy.

The values of the docking energy ΔE of the L-17 compound in the binding sites were −6.6, −8.1, −8.3, −8.1, −7.9, −7.8, −7.4, −8.0, −6.1, −6.3, −7.4, −8.0, −6.5, −6.0 kcal/mol for 5-HT3A, SERT, CHRM1, DRD1, DRD2, DAT, ADRA1, NET, GABA and benzodiazepine sites of GABA-A receptor, GAT1, JAK3, CAPN1 and CFD, respectively. According to the molecular docking data, the L-17 compound had a pronounced multitarget effect. The properties of the holinoblocker were most pronounced in the L-17 compound, which was characterized by the best docking energy in the M1-cholinoreceptor. Dopaminergic properties were also expressed, which were formed from activity against DRD1 and DRD2 and DAT inhibition. The next, most important factor for the compound L-17 was α1-adrenergic blocking activity due to a moderate effect on ADRA1 and a pronounced effect on NET. The moderate serotonin-blocking effect of L-17 was due, above all, to a sufficiently high affinity for SERT; the direct antagonism to 5-HT3A receptors was negligible. The possible presence of a moderate GABAergic effect due to the GAT1 transporter blocking properties was revealed; while the direct interaction with the GABA-binding and benzodiazepine sites of the GABA-A receptor was not significant. There is a high inhibitory activity of the compound L-17 against JAK3 kinase. The inhibition of the other two target proteins, involved in the processes of inflammation and apoptosis (CALPN1 and CFD), was insignificant.

Based on the above results, for a working hypothesis it was suggested that one of the main mechanisms of the anti-inflammatory action of the compound L-17 was the inhibition of JAK3; thus, in the framework of this hypothesis, the in silico of the targeted mechanism of anti-inflammatory action of L-17 was conducted.

#### In Silico Study of the Targeted Mechanism of Anti-Inflammatory Action of Compound L-17

According to the PASS/PharmaExpert predictions and the docking results, the L-17 compound should exhibit Janus tyrosine kinase (JAK) inhibitory activity. It is known that JAKs participate in the signal transduction from the cytokine receptors along the JAK-STAT [77] signaling pathway through the PI3K-AKT signaling pathway [78] and play a key role in the formation of inflammatory processes and the enhancement of apoptosis. Inhibition of JAK3 by pharmacological agents on a myocardial ischemia/reperfusion (I/R) model in mice significantly decreased plasma creatine kinase and lactate dehydrogenase activities, reduced infarct size, reversed I/R-induced functional deterioration of the myocardium, and reduced myocardial apoptosis [79]. Conversely, ischemia/reperfusion, hypoxia, aging, hyperglycemia, inflammation and the oxidative stress accompanying all these processes led to the formation of advanced glycation end-products (AGEs) [80]. AGEs interact with specific AGE receptors (RAGE), which also activate the PI3K-AKT signaling pathway, which in turn leads to the activation of NF-κB [81] and, ultimately, to the increase in inflammation and apoptosis and, possibly, the occurrence of cancer.

To determine the most probable multitarget mechanism of anti-inflammatory action of the L-17 compound, according to the protocol described above, the docking of this compound was performed to specific binding sites of PIK3CA (alpha catalytic subunit of phosphatidylinositide 3-kinase), AKT1 (protein kinase B alpha), RAGE and NF-κB1 (nuclear factor kappa-B p50 subunit). Regarding these four target proteins, optimum experimental X-ray 3D models from Protein Data Bank in Europe [58] with PDB codes2RD0 [82], 5KCV [83], 3O3U [84] and 1SVC [85] for PIK3CA, AKT1, RAGE and NF-kB1, respectively, were found. According to the results obtained, the values of the docking energy ΔE of the L-17 compound in binding sites were estimated as −8.0, −7.3, −8.4, −5.5, −5.8 kcal/mol for JAK3, PIK3CA, AKT1, RAGE and NF- κB1, respectively. Obviously, L-17 did not block RAGE and NF- κB1, but exhibited high affinity for the three kinases JAK3, PIK3CA and AKT1. It can be argued that in this regard, with a fairly high probability, the anti-inflammatory effect of substance L-17: (1) was not related to the signal transduction from RAGE to NF-κB; (2) was due to trinary blocking of kinase signal pathway PI3K-AKT; (3) included the significant immunotropic component.

**Figure 2:**
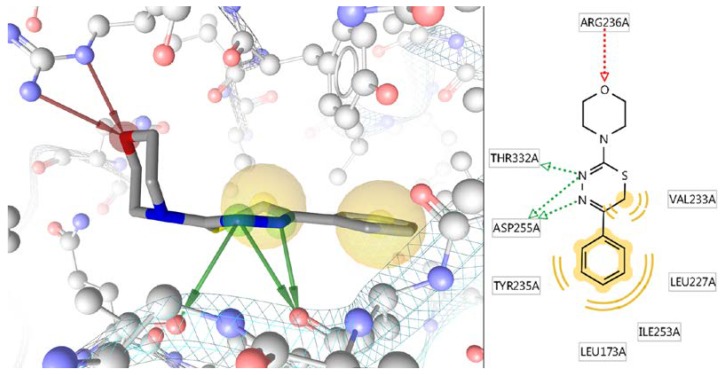
Molecular mechanism of binding of the L-17 molecule to the AKT1 kinase site.

Concerning the most affine to L-17 compound AKT1 kinase with ΔE = −8.4 kcal/mol, an analysis of the molecular binding mechanism was performed using the program Ligand Scout 4.2 Advanced [86] (Figure 2). It is seen that the high binding energy of the L-17 molecule to the AKT1 site was due to 5 hydrogen bonds and two hydrophobic interactions, one of which is very extensive and affects 4 amino acid residues.

### 3.3. In Vivo Studies

#### 3.3.1. Action of Substituted 1,3,4-Thiadiazines on the Course of Stress Reaction

Considering that 1,3,4-thiadiazines revealed effects similar to those of atypical antipsychotics in experiments in vivo, its effects on the course of stress reaction were investigated. The model of immobilization stress used in those experiments was described by Kvetnansky: rats were immobilized fixing their backs to an operation table for 6 h a day for 2 days [87]. Histological, morphological, as well as biochemical studies and immunoassays were performed on the first and second day of the experiment. According to the results obtained, on the background of 3,4-thiadiazines administration there was a significant change in the cellular composition of the blood on the second day: an increased concentration of a mixture of monocytes, eosinophils, basophils, and immature cells in the blood was detected. The glucose level in animals treated with the compounds was significantly lower than in control animals (8.0 ± 0.6 versus. 9.8 ± 0.2) [88].

Since the main target organs under stress are the thymus (stress causes the involution of the thymus lymphatic system) and adrenal glands (hypertrophy of the adrenal cortex), the targeted histological examination of those organs was conducted. According to the results, on the background of L-17 compound administration, the medulla of the thymic lobules retained numerous lymphoid elements, which suggested strengthening of lymphopoiesis [88]. With that, in control animals, thinning of the cortical layer, obliteration of the boundaries between the cortical and medulla layers in the lobules as well as denudation of the stroma of the organ were observed after stress [88]. Moreover, similar changes were described in the in the adrenal glands of control animals: dilated sanguineous blood vessels in the medullary; signs of thromb formation were visible in some; and diffuse extension of vessels at the cortex/medulla boundary [88]. In the pancreas, the consistent increase in the surface area of pancreatic islets and the density of distribution of insulin-producing cells was also observed after L-17 compound administration. This confirms that the L-17 compound influences the stress response because the insulin production is curtailed during the resistance phase and at the start of the depletion phase of stress [89].

Taken together with the results of pharmacological evaluation and behavioral studies, the aforementioned results enabled us to suggest that substituted thiadiazines lower the reaction to stress. With that, the described action of compound L-17 does not contradict the previous data of the pharmacological study and is similar to the action of atypical neuroleptic sulpiride: according to Benelli et al. (2000) treatment with low doses of l-sulpiride lowers the reaction to stress and prevents the development of gastric lesions induced by chronic exposure to uncontrollable stress [90].

Interestingly, according to the literature, drugs acting on the dopaminergic system (including sulpiride), revealed the immunomodulatory effects not only on models of stress damage, but also in such severe diseases as myocardial infarction (MI). According to Tagliavini et al. (1992) specific dopamine antagonists (I-sulpiride) was able to limit ischemia- and reperfusion-induced myocardial damage [91]. Later clinical works showed the beneficial effects of sulpiride on the patients with MI which manifested itself in the decrease in the number of anginal attacks and their severity [92]. Thus, based on the data of the above studies and the similarity of the sulpiride and the L-17 compound according to the analysis of the affinity spectra, the authors were able to assume that the administration of L-17 compound will reduce the stress response and improve the course of stress-related coronary heart diseases and MI [88]. Moreover, several compounds from the group of substituted 1,3,4-thiadiazines (including the L-17 compound) exerted the immunomodulatory properties on experimental models of severe stress and MI.

#### 3.3.2. Action of Substituted 1,3,4-Thiadiazines on the Course of Myocardial Infarction

It should be noted that in cardiovascular diseases, stress damage most often plays one of the main roles in the development of pathological disturbances [93,94], and the heart being the main target for the stress reaction is described in the literature [89,95].

The confirmation of the presence of stress-induced myocardial infarction in the current studies were the levels of aspartate aminotransferase (AST) after immobilization stress, which were significantly higher in the stressed animals in comparison with the control ones and were similar to AST levels in the animals with myocardial infarction (25.6 ± 1.5 U/L after immobilization stress and 24.9 ± 2.0 U/L in 24 h after myocardial infarction) [88]. Modelling of MI in rats was performed in accordance with the authors’ modification of standard ligation model. Histological, biochemical and immunobiochemical studies were performed at days 1, 5, 7 and 14 of the experiment [96,97].

According to the results obtained using L-17 compound administration, significant changes in the histological picture of the heart were observed on the first day of experimental MI. Animals of the control group (with MI induced) had the transmural MI typical for this species [98], which was characterized by the cardiomyocyte’s death in all layers of the heart. The developed MI was located subepicardially, based on L-17 compound administration. The histological picture of the infarction zone on the background of L-17 compound administration was also different: on the first day of experimental MI, the diffuse infiltration of segmented leucocytes which persisted until the 7th day was observed in control animals, while only the lesion zone without a pronounced perifocal exudative reaction was observed after the L-17 compound administration. The decrease in the number of neutrophils observed could cause a decrease in the depth of lesion and the intensity of the inflammatory reaction in experimental MI. The early entry of mononuclear cells (macrophages and lymphocytes) into the damaged tissues, which are necessary for effective resolution of inflammation [99], including the absorption of apoptotic and necrotic cells and the activation of fibroblasts which contributes to the formation of granulation tissue and neoangiogenesis [100], was also detected.

Administration of L-17 compound also led to the earlier and more active reparative process in the affected area. Thus, in control animals, the signs of formation of granulation tissue were revealed only on the seventh day and only in the structures adjacent to the necrotic zone, while on the background of L-17 compound administration, the granulation tissue had appeared in the zone of subacute inflammation by the fifth day of the experiment and had replaced the infarction zone almost completely by the seventh day, with endothelial capillary cells involved in the formation of sinusoidal capillaries, appearing on the fifth day [96,97]. The observed histological picture indicated that the administration of L-17 compound in MI, decreasing the entry of neutrophils into the inflammation zone and increasing the supply of macrophages, revealed the immunomodulatory effect, manifested itself in the change of the exudative-destructive type of inflammation to the proliferative-cellular [96,97].

**Figure 3:**
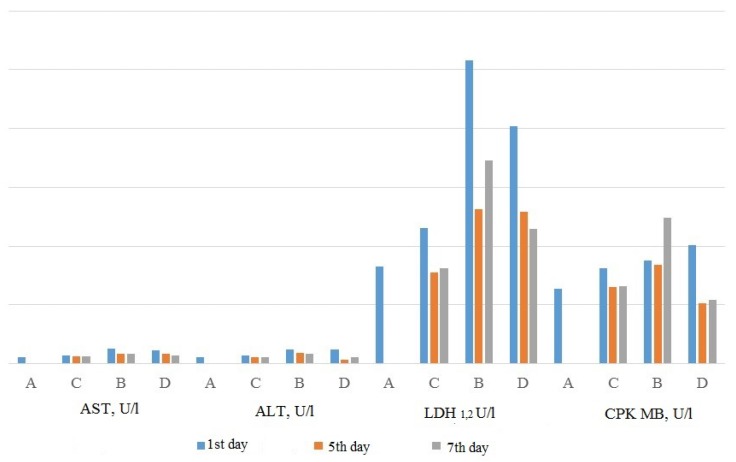
Activity of transaminases in the blood according to the results of the biochemical study.

The results of the biochemical study revealed that the activity of AST, alanine transaminase (ALT) and creatine kinase-MB (CPK MB) in the animals on the background of L-17 compound administration was significantly lower than in the animals of the control group (Figure 3). Moreover, on the fifth day of the experiment, the activity of ALT and the activity of CPK MB did not differ significantly in comparison with intact animals. Concurrently, in animals of the control group with experimental MI, the rise of CPK MB activity on the seventh day exceeding the first day’s activity was detected [96]. Considering that the activity of serum CPK in patients with MI is considered as one of the sensitive diagnostic methods of diagnosing myocardial infarction, the repeated rise of CPK activity in the control group might be considered as the sign of MI recurrence [101].

Additional to the routine evaluation of the activity of enzymes in the blood plasma, their activity in the homogenate of the myocardium was defined [102]. The comparison of lactate dehydrogenase 1,2 (LDH 1,2) and CPK activity in blood and in myocardial homogenate revealed the principal differences between the animals of the experimental (with the L-17 compound administration) and control groups. Thus, on the first day in both groups the decrease in LDH 1,2 activity in the homogenate coincided with its increase in blood, which is typical for the onset of MI, while on the seventh day of the experiment the animals of the experimental group displayed low activity of LDH 1,2 in myocardial homogenate, corresponding to the decrease in activity in the blood, which eliminated the possibility of re-infarction. The animals of the control group showed the repeated increase in LDH 1,2 in the blood on the 7th day of the experiment corresponded to the low enzyme activity in the myocardial homogenate, which confirmed the presence of recurrent MI [71]. Similar changes were observed on the seventh day of the experiment for CPK activity; the activity of CPK in blood corresponded to normal values in myocardial homogenate in the animals of the experimental group, while in the control group the increased activity of CPK MB in the blood, even in comparison with the first days of the experiment, was not accompanied by a change in the activity of CPK MB in the homogenate [102]. Thus, the evaluation of enzyme levels in myocardial tissue confirms previously reported data that the administration of a thiadiazine compound prevents recurrence and decreases the size of experimental MI.

Since the clinical significance of hypercytokinemia in MI is not in doubt, and both the magnitude of the developing MI and the likelihood of complications depend on its severity [103], the levels of pro-inflammatory and anti-inflammatory cytokines (TNF, IL-1, IL-6 and IL-10) were determined [95]. According to the results obtained, at 24 h after MI the levels of interleukins in control group animals exceeded the norm for IL-1 by 8 times, for TNF by 7.8 times, for IL-6 by 2.5 times and for IL-10 in 4,3 times. Concurrently, in the animals of the experimental group with the compound L-17 administration, the increase in interleukin levels was significantly lower: IL-1 was increased 1.8 times, TNF-4.7-fold, IL-10–2.2-fold, and IL-6 level below the norm 2 times [97].

Considering the possibility of the development of systemic inflammation in MI and of objectifying the effects of the revealed hypercytokinemia on the severity of the inflammatory reaction, the values of the reactivity coefficient (CR) and the reactivity level (UR) by Gusev et al. (2008) were calculated [104,105]. The calculations revealed that in the animals of the control group the reactivity coefficient reached 11, which corresponded to fourth level reactivity—the “forecast zone” of critical complications, at which the development of a systemic inflammation process might begin when TNF-α, IL-1β and IL-6 are produced not only by cardiomyocytes, but also by endothelial cells [105,106]. Moreover, on the background of L-17 compound administration, the reactivity coefficient reached nine, which corresponded to the third level of reactivity, typical for the hyperreactive form of classical inflammation, and did not differ from the indices of the sham-operated animals. Thus, one of the main effects of L-17 compound administration in animals with MI was the prevention of its recurrence, confirmed by biochemical and histological data. This effect and the absence of subendocardial damage, can be explained, first, by the changes in the microcirculation state caused by a decrease in the levels of circulating cytokines, which trigger the intravascular coagulation processes [107,108]. Additionally, high levels of circulating cytokines might cause the dose-dependent vasoconstriction of A2–A4 arterioles [109], stimulating local neutrophilia and platelet aggregation [110] and activating procoagulant and prothrombotic processes on endothelial cells [111] which, in turn, cause the disturbances in the microcirculation state.

The decrease in the size of necrosis in experimental MI on the background of L-17 compound administration, indirectly assessed by AST activity, suggested the possibility of the compound’s influence on the cardiomyocytes apoptosis activity. Subsequently, the study showed that on the background of L-17 compound administration, the intensity of apoptosis initiated via extrinsic pathway (CD 95) was significantly higher than in control animals throughout the experiment, while the intensity of apoptosis initiated via intrinsic pathway (P 53) was higher on the seventh day of the experimental MI [112]. The fact that the significantly higher apoptosis activity rates on the background of L-17 compound administration corresponded to significantly lower AST activity, in comparison with the control animals, indicated the reduction in the intensity of cell death by necrosis at the same time.

## 4. Discussion

### The Proposed Mechanisms of Action of Substituted 1,3,4-Thiadiazines

**Figure 4:**
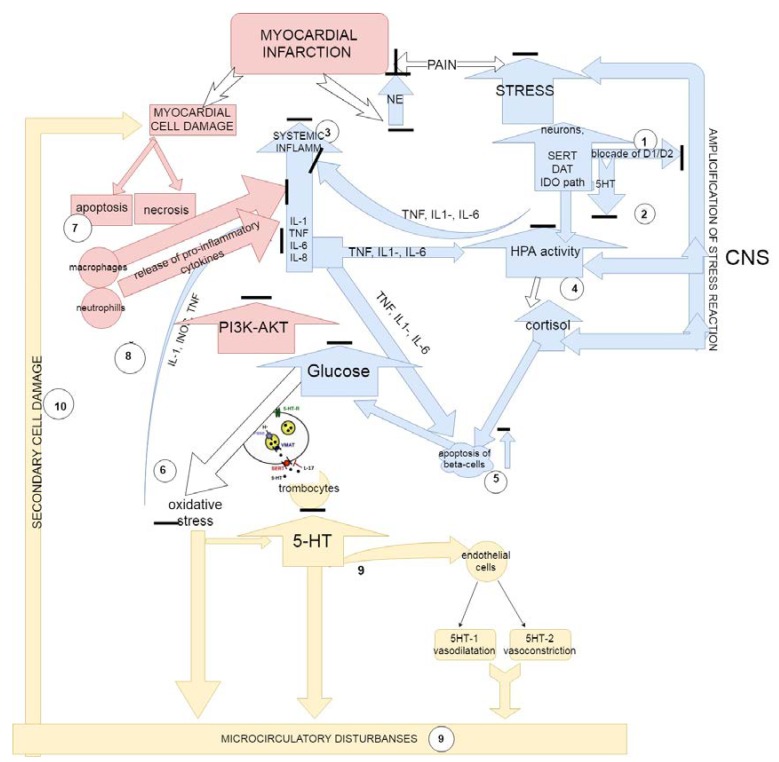
The proposed immunopathophysiological mechanisms of stress-limiting effects of 1,3,4-thiadiazine compounds in myocardial infarction (based on the compound L-17 properties). Legend: blue—the main mechanisms of action of the substituted 1,3,4-thadiazines on the course of stress reaction are highlighted; red—mechanisms acting on the tissue damage; yellow—action of the compounds on the serotonin-mediated disorders of microcirculation. Systemic effects of 1,3,4-thadiazines included: (1) the increase in neuronal stress-resistance mediated through D2 receptors; (2) the increase in synaptic availability of 5-HT, mediated via SERT and M1 receptors; (3) the reduction in the levels of stress cytokinemia, mediated via NET, M1 and D2 receptors; (4) the reduction of stress hormonal response and sensitivity of pituitary-adrenal system mediated through blockade of M1 receptors; (5) the reduction of stress-mediated decrease in insulin production in the pancreas and prevention of stress hyperglycemia due to action on dopamine receptors; (6) the decrease in of blood glucose levels, which, on the background of M-cholinoreceptor blockade, led to the decrease inoxidative stress in tissues. Local effects of substituted 1,3,4-thadiazines included: (i) the reduction of oxidative stress levels and the suppression of the intensity of the local inflammatory process by inhibiting chemotaxis and migration of leukocytes, which was mediated through the blockade of muscarinic receptors and the kinase signal pathway PI3K-AKT; (7) the increased apoptosis, increased macrophage infiltration, decreased macrophage release of proinflammatory cytokines via blockade of D-receptors; (8) the decreased platelet activation and aggregation preventing the development of microcirculatory disorders and, as a consequence, secondary damage to the myocardial tissues (9–10) through a synergistic blockade of the serotonergic and adrenergic systems.

The revealed cardioprotective effects of substituted thiadiazines might be explained by the peculiarities of their multi-target action: the ability of the compounds to interact with various types of receptors and transporters of dopaminergic, serotonergic and acetylcholinergic systems and to block the kinase signal pathway PI3K-AKT (Figure 4). Since the activity of these systems makes a significant contribution to the pathogenesis of MI, the complex effect on them leads to significant changes during the pathological process and determines the occurrence of the described cardioprotective effects. Thus, the multi-target action of thiadiazine compounds affected both the manifestations of the stress reaction and the course of developed MI.

One can propose that, on the basis of thiadiazines administration, there occurred an increase in the stress-resistivity of neurons (action through D2 receptors) [113] and a decrease in the levels of stress cytokinemia (from critical levels, observed after MI) [114,115], which was mediated through SERT [17,113] and central M1 receptors [113] and was accompanied by the decrease in the stress hormonal response due to the blockade of M1 receptors [116], a stress-mediated decline in insulin production in the pancreas and the occurrence of stress hyperglycemia due to the action on the dopamine system [90,117]. Reduction of the peripheral organs (thymus, adrenal glands) reaction under stress was due to action of the compounds through NET, M1 and D2 receptors [118,119].

Substituted thiadiazines in the myocardium, acting through the D-receptors, intensified the apoptosis processes and increased macrophage infiltration [120], thereby having reduced the release of pro-inflammatory cytokines by the latter and lymphocytes. The blockade of muscarinic receptors led to the suppression of the intensity of the local inflammatory process due to inhibition of chemotaxis and the migration of leukocytes [121], which led to the reduction in leukocyte infiltration and tissue damage [122].

Synergistic effects of serotonergic [123,124], adrenergic [125] and cholinergic [126] system blockades on the processes of platelet activation and aggregation prevented the development of microcirculatory disorders which contribute significantly to the recurrence of MI. Moreover, the L-17 compound’s ability to block kinase signal pathway PI3K-AKT, predicted in in silico studies, also might be responsible for the appearance of the described effects on experimental MI since, according to the literature, the inhibition of JAK3 significantly decreases the plasma creatine kinase and lactate dehydrogenase activities and reduces the infarct size [79]. Consequently, in the animals with experimental MI, using 1,3,4-subsituted thiadiazine administration, the described pattern was noted, characterized by a less pronounced cytokinemia and stress increase in glucose, a less pronounced involution of target organs, in the myocardium with smaller MI dimensions [59], and a decrease in the frequency of its recurrence accompanied with the change of the inflammation type to a productive one.

## 5. Conclusions

While the stress response and the immune systems are different parts of the larger defensive system [10], some might discover some immunomodulatory effects, and vice versa. This concept was confirmed by the series of studies [19,20,21,22,23,24,25,26,27,28,29] and found confirmation in the current studies [96,97,112]. Thus, it was found that several chemical compounds of the group of substituted thiadiazines possessed the attributes of atypical antipsychotics, having a significant effect on the behavior of animals and the course of the stress reaction [54,88]. Concurrently, this class of compounds had an exceptional breadth on the biological activity spectrum [38,39,40,41,42,43,44,45,46,47,48,49,50,51,52]. Such pleiotropic actions served as the basis for the search for a single mechanism capable of influencing both the course of the inflammatory process and the stress reaction.

According to the results of in vivo experiments, the immunomodulatory cardioprotective effects of substituted 1,3,4-thiadiazines in MI, expressed in the reduction of MI size (the formation of only a subepicardial infarction) and the prevention of its recurrence, were due to their action not only in the ischemic tissues but, also, on the severity of the stress reaction [96,97].

The study cycle conducted in silico allowed revelation of the main targets regarding the action of compounds within several 1,3,4-thiadiazines which included receptors and transporters of serotonergic, adrenergic and dopaminergic systems. Additionally, the potentially high activity of chemical compounds on the blockade of JAK3 was determined. Moreover, if the influence of the activity of the compound upon the neurotransmitter systems on the course of the stress reaction is understandable, the effect of the blockade of the kinase signal pathway PI3K-AKT requires further research. The possibility of such influence has already been described: some anti-depression drugs that were used in anatomical models of “sickness behavior” and in human depression clinical trials were shown to suppress the clinical markers of inflammation, as well as SAPK/MAPK and/or JAK/STAT signaling in vitro [127]. Later, the experimental work of Jiang et al. (2017) also revealed that antidepressant-like effects of magnesium isoglycyrrhizinate and fluoxetine might be mediated by the JAK/STAT/NF-κB signaling pathway [128].

Thus, the described effects of substituted 1,3,4-thiadiazines convinced the authors of the need to search for a new agents for limiting the peripheral inflammatory/ischemic damage through the central mechanisms of stress reaction and modifying pro-inflammatory cytokine signaling pathways in the brain.

## Figures and Tables

**Table 1:** Chemical structures of substituted 1,3,4-thiadiazines, mentioned in the text.

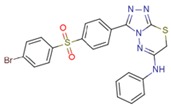	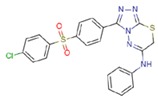	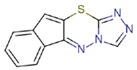
Reaxys RN = 20515191 (Ref. [38])	Reaxys RN = 20515189 (Ref. [38])	Reaxys RN = 21926455 (Ref. [40])
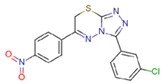	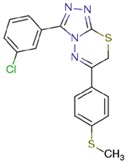	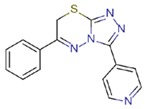
Reaxys RN = 6006308 (Ref. [41])	Reaxys RN = 22412039 (Ref. [41])	Reaxys RN = 5094636 (Ref. [45])
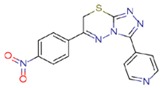	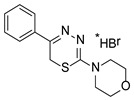	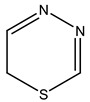
Reaxys RN = 6535400 (Ref. [45])	L-17, a 5-phenyl substituted-6H-1,3,4-thiadiazine-2-amine. (Ref. [48,49,50,51,52,53,54])	1,3,4-thiadiazine cycle

**Table 2:** Chemical structure of compounds CHEMBL2104951 and CHEMBL2440857.

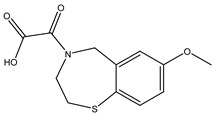	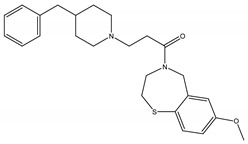
CHEMBL2104951	CHEMBL2440857

**Table 3:** Biological action of the compounds CHEMBL2104951 and CHEMBL2440857.

ChEMBL ID, Name, Code	Assay Description	Activity ID	Activity
CHEMBL2104951 Aladorian ARM036 S36	Induction of cardiac function improvement in myocardial infarction-induced acute heart failure mouse model at plasma concentration 200 nM after 7 days post myocardial infarction.	16878971	Active
	Induction of cardiac function improvement in myocardial infarction-induced acute heart failure mouse model subjected to permanent ligation of left anterior descending coronary artery assessed as increase in fractional shortening at plasma concentration of 100 to 200 nM after 2 weeks post-myocardial infarction by echocardiography.	16878972	Active
	Induction of cardiac function improvement in myocardial infarction-induced acute heart failure mouse model subjected to permanent ligation of left anterior descending coronary artery assessed as increase in cardiac contractility at plasma concentration 200 nM after 7 days post-myocardial infarction.	16878973	Active
CHEMBL2440857 JTV-519	Reduction in PKA-phosphorylation of RyR2 in myocardial infarction-induced heart failure mouse model at 0.5 mg/kg/h after 28 days post-myocardial infarction.	16878976	Active
	Improvement in soleus muscle fatigability in myocardial infarction-induced heart failure mouse model at 0.5 mg/kg/day relative to control.	16878983	Active
	Improvement in soleus muscle fatigability in myocardial infarction-induced heart failure calstabin2-deficient knockout mouse at 0.5 mg/kg/day relative to control.	16878984	Active
	Normalization of RYR1 in myocardial infarction-induced heart failure mouse soleus muscle assessed as increase in average channel open dwell time.	16878985	t = 1.5 ms
	Normalization of RYR1 in myocardial infarction-induced heart failure mouse soleus muscle assessed as decrease in average channel close dwell time.	16878986	t = 1567 ms
	Induction of calstabin-1 binding to RYR1 in myocardial infarction-induced heart failure wild type mouse soleus muscle at 0.5 mg/kg/day dosed via implantable osmotic minipumps.	16878996	Active
	Inhibition of of PKA-induced RyR1 phosphorylation in myocardial infarction-induced heart failure mouse soleus muscle at 0.5 mg/kg/day dosed via implantable osmotic minipumps.	16878997	Active
	Induction of calstabin-1 binding to RYR1 in myocardial infarction-induced heart failure calsiabin2^−/−^ mouse soleus muscle at 0.5 mg/kg/day dosed via implantable osmotic minipumps.	16878998	Active
